# Supplementary material for: Benefits of NGS in Advanced Lung Adenocarcinoma Vary by Populations and Timing of Examination
Source: Int J Mol Sci. 2024 Jun 25;25(13):6949. doi: 10.3390/ijms25136949 (PMC11241057; doi:10.3390/ijms25136949)
Supplement: Supplementary file 1 [file ijms-25-06949-s001.zip › ijms-3050485 Supplementary.pdf]

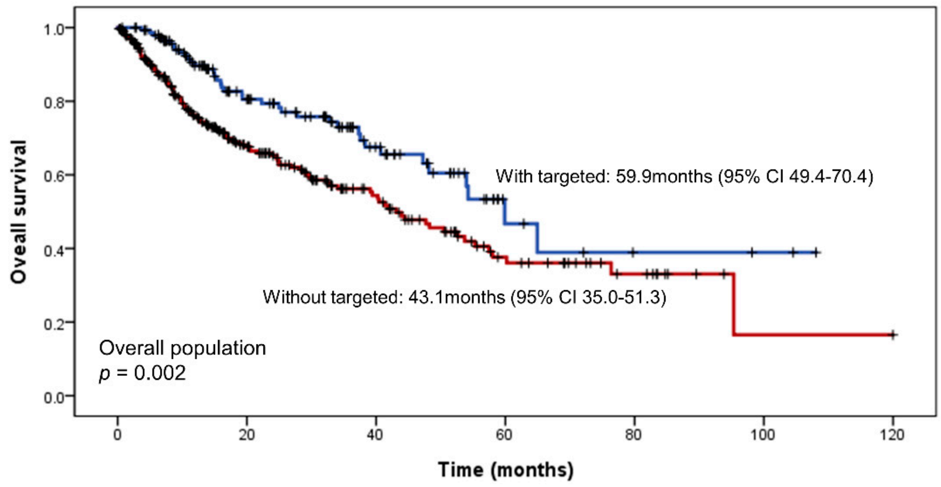


Figure S1. Survival benefits of next-generation sequencing and mutation-targeted treatment in the overall population.

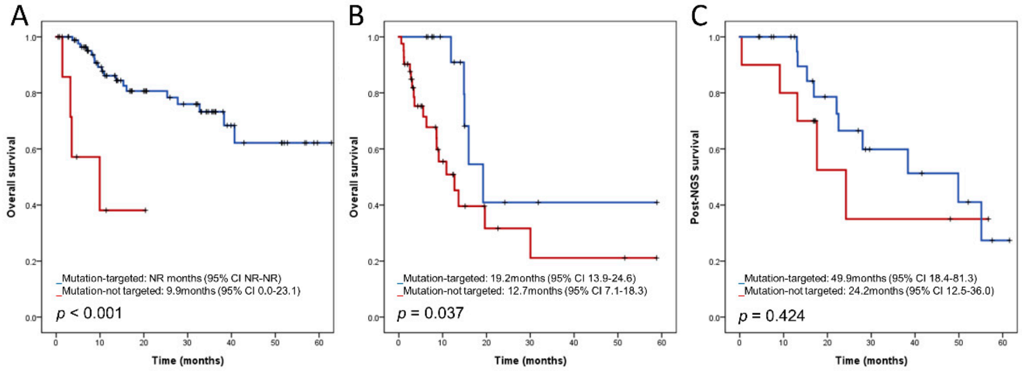


Figure S2. Subgroup survival analysis of (A) Group 1, (B) Group 2, and (C) Group 3 patients among those with targetable mutation(s).

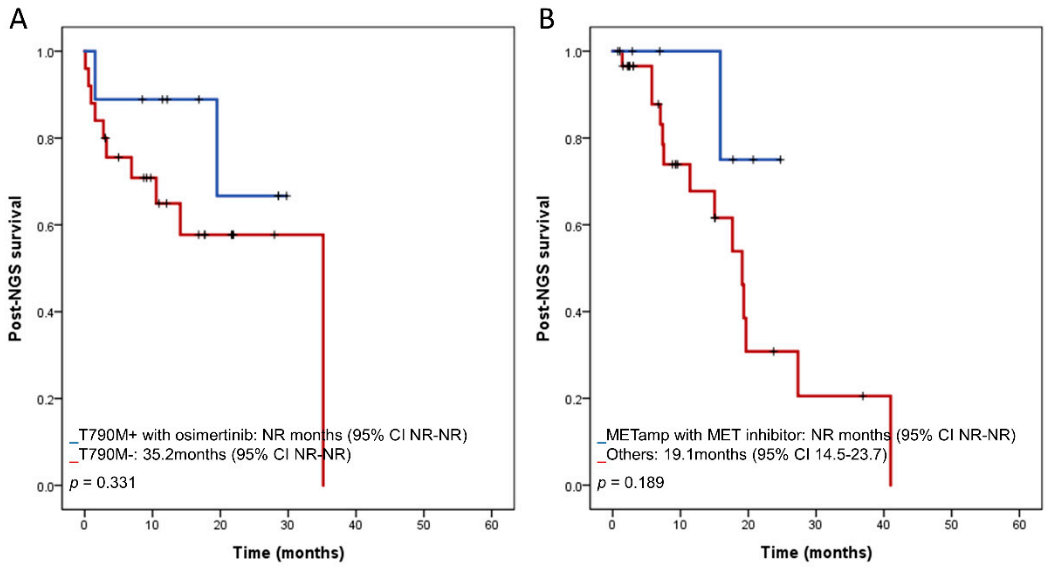


Figure S3. Subgroup survival analysis of Group 4 patients with progression on first- or second-generation EGFR-TKI (A) and progression on osimertinib (B)

Supplemental Table S1. Comparison of baseline characteristics between patient groups

| | Group 1 | Group 2 | Group 3 | Group 4 | P value* |
|---|---|---|---|---|---|
| Age, ≥ 65 years, n (%) | 43 (33.6) | 67 (53.2) | 42 (46.7) | 21 (26.3) | <0.001 |
| Gender, female, n (%) | 68 (53.1) | 48 (38.1) | 42 (46.7) | 50 (62.5) | 0.005 |
| Smoking, smokers, n (%) | 44 (34.4) | 75 (59.5) | 39 (43.3) | 25 (31.3) | <0.001 |
| Tumor stage, IV, n (%) | 123 (96.1) | 121 (96.0) | 85 (94.4) | 79 (98.8) | 0.526 |
| ECOG PS, 0-1, n (%) | 117 (91.4) | 95 (75.4) | 85 (94.4) | 74 (92.5) | <0.001 |
| Specimen, blood, n (%) | 61 (47.7) | 32 (25.4) | 76 (84.4) | 43 (53.8) | <0.001 |

ECOG PS, Eastern Cooperative Oncology Group performance status

*By Fisher's exact test.

Supplemental Table S2. Next-generation sequencing platform used in the present study

| Specimen types | NGS platforms | Patient number (%) |
|---|---|---|
| Tumor tissue | FoundationONE® CDx | 96 (22.6%) |
| | Oncomine™ Focus Assay | 45 (10.6%) |
| | ACTDrug®+ | 39 (9.2%) |
| | ACTOnco®+ | 31 (7.3%) |
| Liquid | GUARDANT360® | 181 (42.7%) |
| | ACTMonitor® Lung | 13 (3.1%) |
| | ACT Cerebra™ | 8 (1.9%) |
| | FoundationONE® LIQUID CDx | 6 (1.4%) |
| | TruSight™ Oncology 500 ctDNA | 4 (0.9%) |
| | SOFIVA Cancer Monitor | 1 (0.2%) |
